# On the Employment of Castor Seeds

**Published:** 1845-01

**Authors:** 


*On the Employment of Castor Seeds.*—Castor oil, says M. Soubeiran, is less purgative than the seeds which furnish it. This is because the oil which flows out in the press contains, comparatively, less resin than remains in the residue. M. Mialhe relates various therapeutical results obtained by means of an emulsion, prepared with fresh seeds of castor, which entirely confirmed this opinion; for, with ten grammes of seeds deprived of their shells, there was an emeto-cathartic effect, which continued for three days, neither opiates, cold gaseous drinks, nor cataplasms being able to subdue it. An emulsion prepared with six grammes produced twenty-eight vomitings, and eighteen alvine evacuations. Finally, with a third emulsion, containing only one grain of castor seeds, the emeto-cathartic effect was still very powerful. From these facts M. Mialhe concludes: 1st. That the oleoresinous principle found by M. Soubeiran in castor seeds exists only in very small proportion in the oil of these seeds, whilst the whole of it was found in their emulsion. 2d. That French castor contains a large proportion of the acrid emeto-cathartic principle belonging to a great number of the plants of the *Euphorbiaceæ.* 3d. That the emulsion of castor seeds prepared with only twenty, thirty, or fifty centigrammes of these seeds, constitutes, perhaps, the most agreeable purgative of all those at present in use, (if, however, the vomitive effect of this emulsion completely ceases when the dose of seed is suitably diminished.) Although this latter peculiarity has not yet been proved by clinical observation, it is probable that it is the case; for it is almost certain that the active principle of castor is analogous to, if not identical with, that of croton oil. Now, it is known that this latter oil, which is a simple purgative in the dose of one drop, becomes emeto-cathartic when this small dose is exceeded.—*Boston Med. and Surg. Jour.*, from *Bulletin de Therapeutique.*

